# Conceptual energy and water recovery system for self-sustained nano membrane toilet

**DOI:** 10.1016/j.enconman.2016.07.083

**Published:** 2016-10-15

**Authors:** Dawid P. Hanak, Athanasios J. Kolios, Tosin Onabanjo, Stuart T. Wagland, Kumar Patchigolla, Beatriz Fidalgo, Vasilije Manovic, Ewan McAdam, Alison Parker, Leon Williams, Sean Tyrrel, Elise Cartmell

**Affiliations:** a Combustion and CCS Centre, Cranfield University, Bedford, Bedfordshire MK43 0AL, UK; b Offshore Renewable Energy Engineering Centre, Cranfield University, Bedford, Bedfordshire MK43 0AL, UK; c Bioenergy and Resource Management Centre, Cranfield University, Bedford, Bedfordshire MK43 0AL, UK; d Power Engineering Centre, Cranfield University, Bedford, Bedfordshire MK43 0AL, UK; e Cranfield Water Sciences Institute, Cranfield University, Bedford, Bedfordshire MK43 0AL, UK; f Competitive Creative Design Centre, Cranfield University, Bedford, Bedfordshire MK43 0AL, UK

**Keywords:** Thermochemical conversion, Energy recovery, Non-sewered sanitary systems, Nano Membrane Toilet, Process modelling, Reinvent the Toilet Challenge

## Abstract

With about 2.4 billion people worldwide without access to improved sanitation facilities, there is a strong incentive for development of novel sanitation systems to improve the quality of life and reduce mortality. The Nano Membrane Toilet is expected to provide a unique household-scale system that would produce electricity and recover water from human excrement and urine. This study was undertaken to evaluate the performance of the conceptual energy and water recovery system for the Nano Membrane Toilet designed for a household of ten people and to assess its self-sustainability. A process model of the entire system, including the thermochemical conversion island, a Stirling engine and a water recovery system was developed in Aspen Plus®. The energy and water recovery system for the Nano Membrane Toilet was characterised with the specific net power output of 23.1 Wh/kg_settledsolids_ and water recovery rate of 13.4 dm^3^/day in the nominal operating mode. Additionally, if no supernatant was processed, the specific net power output was increased to 69.2 Wh/kg_settledsolids_. Such household-scale system would deliver the net power output (1.9–5.8 W). This was found to be enough to charge mobile phones or power clock radios, or provide light for the household using low-voltage LED bulbs.

## Highlights

• Energy and water recovery system from human excreta is modelled in Aspen Plus.
• The Nano Membrane Toilet is proven to be a self-sustained system.
• Up to 87% of total amount of water fed to the system can be recovered.
• Net power output of the entire system is similar to the USB port peak power (2–6 W).
• The specific net power output varies between 23.1 and 69.2 Wh/kg_settledsolids_.

## 1 Introduction

Although 2.1 billion people have gained access to improved sanitation facilities, which are likely to safely separate human excreta from human contact, since 1990, 2.4 billion people still use unimproved, shared facilities or practice open defecation [1]. It has been estimated that around 18% of urban population and 49% of the rural population still lacks access to the health benefits that improved sanitation facilities can provide [1], [2]. This is because the conventional flush toilet, which is widely utilised in the developed countries, is a cost-, resource- and energy-intensive system. A requirement of collection, transportation, treatment and waste disposal processes, which require human resources, chemicals and water, among the others, imposes a considerable financial and environmental burdens on the urban and rural communities [3]. Poor sanitation systems lead to sewage and untreated residuals being released to the environment, which are often discharged directly into seas and rivers, and could infiltrate to the groundwater leading to pollution the surface and ground waters [4], [5], [6], [7]. More importantly, open defecation and discharge of untreated residuals impose a significant hazard to human health in the immediate living environment [3], [8]. It has been estimated that each year diarrhoea kills 1.4 million people [9] that could have been prevented through safe drinking water and ensuring adequate sanitation and hygiene [10]. For these reasons, revolutionary sanitary solutions enabling cost-efficient, human-safe and environmentally-friendly utilisation of human excreta need to be developed [11].

To improve access to affordable, safe and sustainable sanitation, the Reinvent the Toilet Challenge was established by the Water, Sanitation and Hygiene programme of the Bill & Melinda Gates Foundation. It aims to develop low-cost toilets for treating human excreta and recovering useful resources [2], [11], [12] without producing hazardous products. Recent studies performed by Onabanjo et al. [3], and Monhol and Martins [13] have proven that gasification and combustion are feasible thermochemical conversion processes to utilise the chemical energy of the settled solids from human excreta, which comprises wet faecal matter, regardless of their high moisture content. Moreover, Liu et al. [14] have proposed the self-sustained system for the settled solids from human excreta to power conversion that utilises plasma gasification and solid oxide fuel cells. Such a system was found to have the specific net power output of 194.4–357.3 Wh/kg_settledsolids_, depending on whether the faecal matter was dried or not prior to plasma gasification. Alternatively, Parker [12] proposed a novel toilet concept that utilises a nano membrane for water recovery from supernatant, which is mainly composed of urine, and for energy recovery from settled solids to make the system self-sustainable. The thermodynamic performance of this concept has not been yet evaluated.

Considering the fact that the settled solids from human excreta were proven to be a viable energy source [3], [13], this study proposes a conceptual energy and water recovery system for the Nano Membrane Toilet to evaluate the thermodynamic viability of the toilet concept proposed by Parker [12]. The proposed system is designed to process the human excreta from a household of ten people, considering the average input rates of the wet faecal matter and urine of 200 g per capita per day [15], [16] and 1.5 dm^3^ per capita per day [17], [18], respectively. Using the equilibrium approach, which is commonly applied for modelling of thermochemical conversion of solid fuels [19], [20], [21], [22], [23], and employing the pseudo Stirling engine model in Aspen Plus®, the process performance is evaluated in terms of net power output, specific net power output, and water recovery rate. These parameters are considered as the key performance indicators for the entire system. Finally, the parametric study is performed on the main design parameters of the entire system under power and water priority modes, to analyse the system behaviour and to maximise the process performance.

## 2 Model development

### 2.1 Process description

**Fig. 1:**
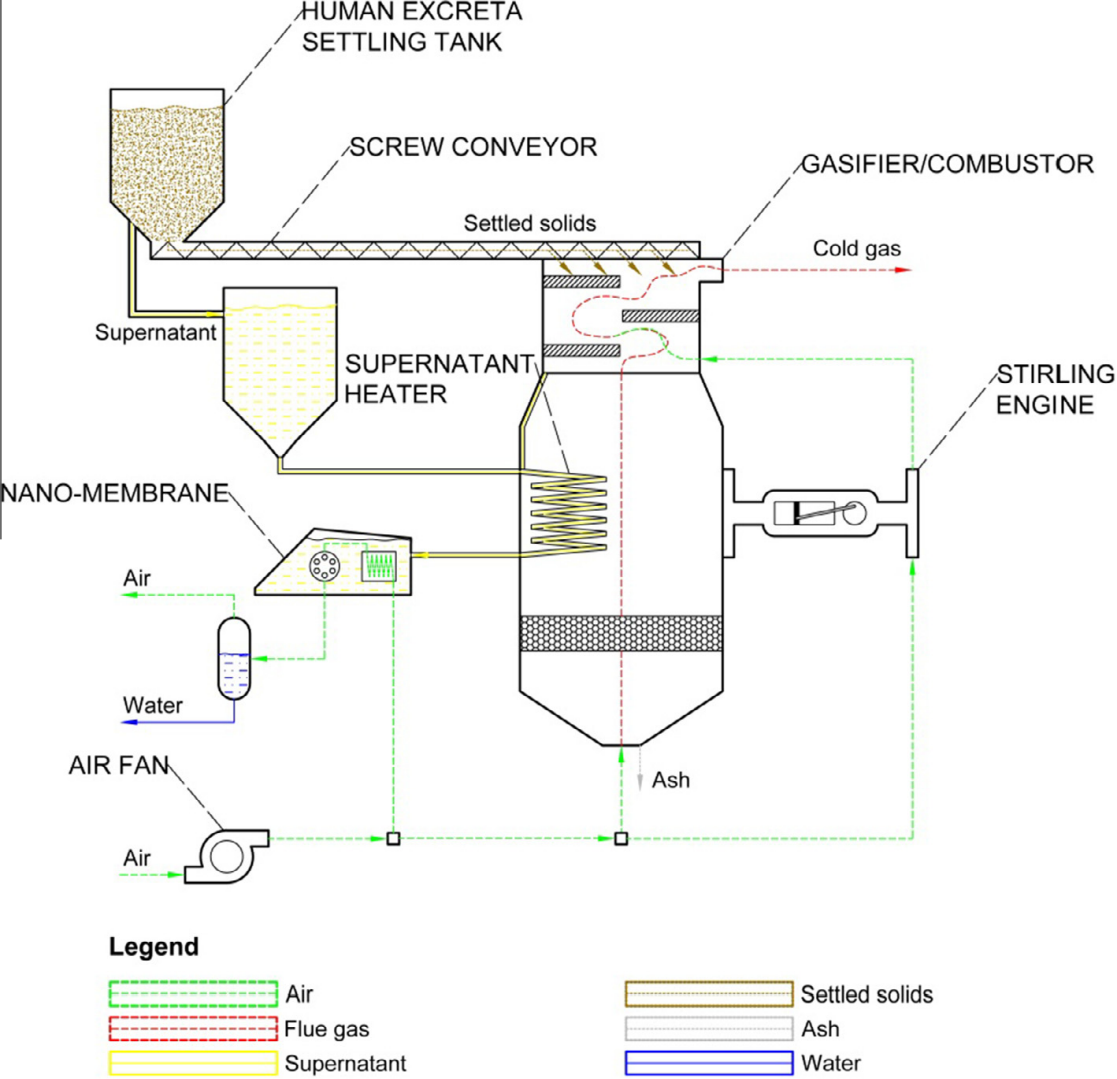
Schematic representation of the conceptual energy and water recovery system for the Nano Membrane Toilet.

The energy and water recovery system proposed in this study (Fig. 1) aims at recovering chemical energy from the settled solids from human excreta, which comprises wet faecal matter, and water from supernatant, which is urine, unbound and partially-recovered bound water from wet faecal matter. Although unbound water and urine can be separated from human excreta in the settling tank, the settled solids transferred to the reactor using a mechanical screw conveyor still comprise around 75–80 wt% moisture [12]. A further reduction of the moisture content to the level that is comparable to other kinds of biomass is achieved on drying the settled solids directly against the hot gas leaving the reaction chamber. It is expected that part of the bound water can be recovered during the drying process that is then mixed with the supernatant stream leaving the settling tank. Dried solids are then fed to the reactor that, depending on the amount of air fed to the reactor represented by an equivalence ratio (ER), operates under gasification (ER < 0.5) or combustion (ER ⩾ 0.5) regime [24], [25], [26], [27], [28]. Therefore, the chemical energy of the settled solids from human excreta can be converted to either chemical energy of synthetic gas that can be utilised in an internal-combustion engine or thermal energy for recovery in an external-combustion engine. In this study, it is conceptualised that a hot-site of the Stirling engine, which is the external-combustion engine known for quiet operation and ability to utilise even low-grade heat [29], is attached to the reactor wall and the cold-side is cooled with part of air leaving the air fan. It is predicted that such concept has a potential to generate sufficient amount of electricity for the entire system to become self-sustained. However, it needs to be highlighted that the amount of energy recovered by the Stirling engine is not only limited by the desired moisture content and temperature of solids leaving the dryer, it also depends on the amount of supernatant that needs to be preheated to the desired temperature for an optimum water recovery in the membrane.

### 2.2 Model description

The steady-state process model for the conceptual energy and water recovery system for the Nano Membrane Toilet developed in Aspen Plus® comprises two main components – the thermochemical conversion island, and the energy and water recovery island – and assumes an ideal behaviour of gases.

In the thermochemical conversion island, which was successfully validated by Onabanjo et al. [3], solid transport and thermochemical conversion are modelled using solids handling features available in Aspen Plus® [30]. Transport of settled solids is conducted using the screw conveyor. Whereas it can be powered through human endeavour, this study assumes the screw conveyor, which has a conservative specific power requirement of 0.056 Wh/kg_settledsolids_ [31], [32], is electrically driven by power generated in the Stirling engine. To accurately account for the composition of the settled solids, these are modelled as a nonconventional component with proximate and ultimate analysis provided in Table 1, and heating value determined from its composition using Dulong’s formula.

The drying process is modelled as a stoichiometric reactor, in which the conversion of bound water to unbound water in settled solids is determined from the dryer material balance using the calculator block, with the desired moisture content of dried solids as an input parameter. By considering the flash separator in the dryer model, it is determined whether the amount of energy carried with hot gas entering the dryer is sufficient to remove desired amount of moisture.

Assuming that the residence time is sufficient for the system to reach chemical equilibrium, which is a common assumption in investigating the thermochemical conversion of solid fuels [19], [20], [21], [22], [23], a thermochemical conversion of dried solids is modelled using the Gibbs reactor, in which Gibbs free energy is minimised to determine the equilibrium composition of the product gas at given operating conditions. Yet, the Gibbs free energy cannot be calculated for dried solids modelled as a nonconventional component. Therefore, the Gibbs reactor is preceded by the yield reactor that is used to model the biomass decomposition into its constituents, for which the Gibbs free energy can be estimated. Importantly, to account for the heat of biomass decomposition, both reactors are connected with the heat stream. The operating regime of the Gibbs reactor (gasification or combustion) is determined by the amount of air fed via the air fan, which is modelled as a compressor with isentropic and mechanical efficiencies of 90% and 99.8%, respectively [34], [35]. The air fan is employed to increase the air pressure by 15 mbar to account for the pressure drop throughout, and thus to reliably estimate the energy requirement of the entire energy and water recovery system.

The energy and water recovery island involves a heat exchanger network, in which the heat exchangers for air and supernatant (Table 1) preheating are modelled as counter-current heat exchangers characterised with a fixed temperature approach and a pressure drop of 5 mbar, the low glass-transition temperature hollow-fibre membrane that recovers water from the supernatant, and the Stirling engine that converts thermal energy to electricity. In the proposed concept, the energy requirement of the membrane stems from the power requirement to increase the pressure of sweep air pressure to overcome the pressure drop in the membrane, which is assumed to be 4 mbar in all evaluated cases, and the heat requirement to preheat the supernatant to 60 °C. As the permeability and selectivity of the membrane has not been yet quantified, a simplified approach to membrane modelling is employed in this conceptual study. Namely, water is partially removed from the preheated supernatant in a component separator and then mixed with the preheated sweep air. Considering low-temperature operation of the membrane, and thus possible condensation of water, the membrane separation efficiency is determined in a calculator block to arrive at 80% vapour fraction in the retentate (Table 2). Water is then recovered from the retentate in a flash separator.

**Fig. 2:**
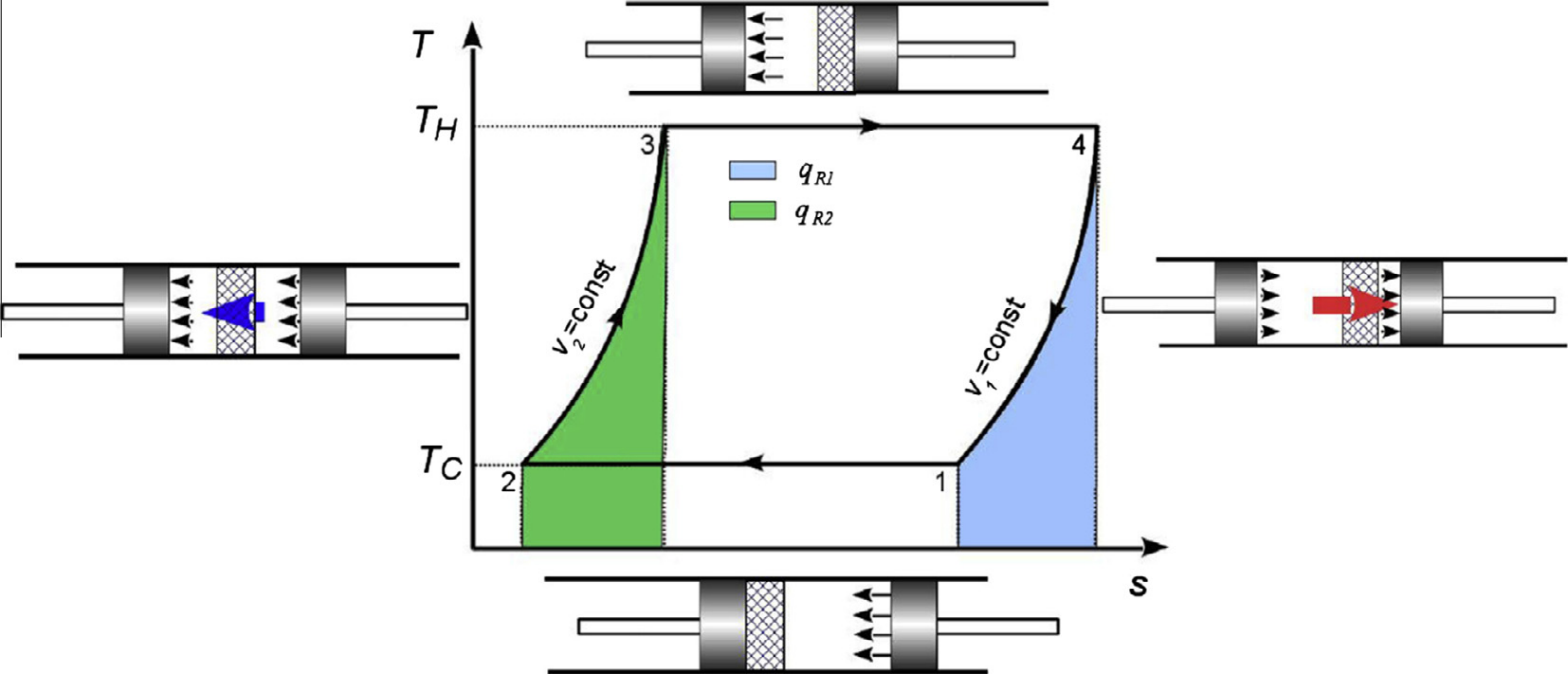
Operating principle of ideal Stirling engine [36].

The ideal Stirling cycle (Fig. 2) comprises four processes: isothermal compression of working medium (helium) with heat release into the heat sink (1 → 2); isochoric heating of working medium in the regenerator (2 → 3); isothermal expansion of working medium with heat gain from the heat source (3 → 4); and isochoric cooling of working medium in the regenerator (4 → 1) [37]. Yet, the Stirling engine model is based on a pseudo Stirling cycle, as it is regarded to provide a closer approximation of the actual engine performance compared to the ideal Stirling engine [29]. The considered model utilises, therefore, the isentropic compressor and expander, as opposed to isothermal compression and expansion in the ideal Stirling engine, what allows accounting for the irreversibility in the process. Moreover, the heater, cooler and regenerator are modelled with a fixed approach temperature difference, as opposed to the infinite surface are in the ideal Stirling engine [29]. The initial design parameters of the entire process, including those used in the Stirling engine model, are reported in Table 2.

## 3 Process analysis

### 3.1 Considerations

Having modelled the entire energy and water recovery system for the Nano Membrane Toilet in Aspen Plus®, its performance is evaluated in terms of the system’s net energy balance, which would indicate whether the system can become self-sustained, and the system’s ability to recover water. The key performance indicators considered in this study are net power output, which refers to the excess power available after the systems’ power requirements are satisfied, specific power requirement and water recovery rate. Nevertheless, evaluation of the concept only under initial design parameters may not allow drawing relevant and accurate conclusions regarding the system’s performance, especially in light of early stage of the concept development and lack of similar concepts in the literature. For this reason, to evaluate the effect of the design parameters on the system’s performance, the parametric studies are conducted by varying:

• desired moisture content of dried solids between 10 and 60 wt%;
• equivalent ratio between 0.7 and 1.2;
• mass flow rate of sweep air to membrane between 1 and 70 kg/day;
• fraction of gas to Stirling engine between 0.1 and 0.6;
• maximum temperature of working medium in Stirling engine between 500 and 640 °C;
• Stirling engine heater approach temperature between 10 and 150 °C;
• Stirling engine cooler approach temperature between 10 and 50 °C;
• Stirling engine cooling air approach temperature between 10 and 50 °C;

The parametric analysis will support selection of the design parameters that will maximise both energy and water recovery in the Nano Membrane Toilet. The analysis is performed with an assumption that the system has capacity to process the excreta from the household of ten people.

### 3.2 Performance evaluation

A performance analysis of the energy and water recovery system for the Nano Membrane Toilet confirmed that under the initial design basis presented in Table 2, the system can become self-sustained and, in addition, partially recover water from supernatant.

As reported in Table 3, for the system capacity to process excreta from the household of ten people, the net power output of the Stirling engine would be 1 W, which corresponds to the specific net power output of 12.3 Wh/kg_settledsolids_. Moreover, the efficiency of the Stirling engine, which is defined as the ratio of the electric output and the heat input in the heater, was estimated to be 19.7%, which is in agreement with values reported in the literature for similar operating temperatures [38], [39], [40], [41]; hence proving validity of the modelling approach. Having assumed that any excess power, is utilised to drive the membrane, it was determined that 54.9 kg/day of the sweep air can be fed to the membrane for the system to remain self-sustained. Yet, for the conceptual system operating under such conditions the water recovery rate was 8.8 dm^3^/day. This corresponds to 57.1% of the total amount of water fed to the system that comprises both the bound water in the settled solids and the unbound water in the supernatant. By considering the results obtained for the system operating under initial design conditions as a reference, further optimisation of the design parameters is undertaken to maximise both net power output, which can be utilised to power some of the household devices or during the start-up of the system, and water recovery.

### 3.3 Parametric study

#### 3.3.1 Thermochemical conversion island

**Fig. 3:**
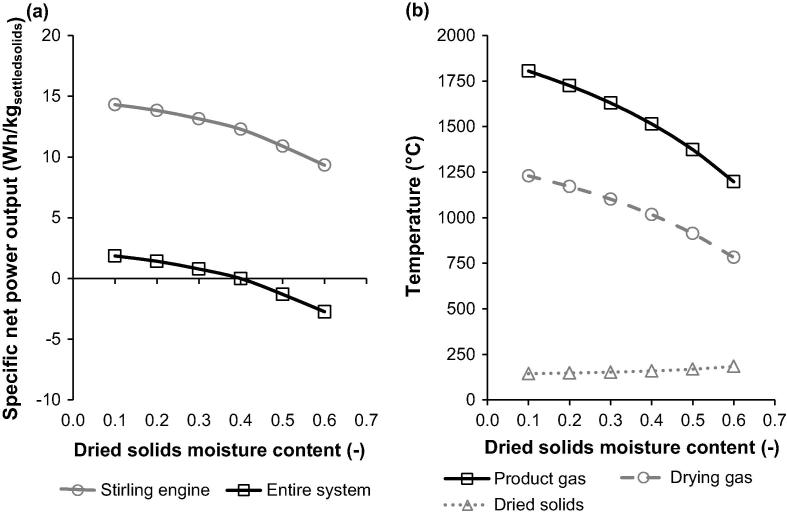
Effect of dried solids moisture content on specific net power output and temperatures throughout the energy and water recovery system.

The performance of the conceptual energy and water recovery system proposed in this study is directly dependent upon amount of energy recovered from the settled solids from human excreta in the thermochemical conversion island. As indicated in Section 2.1, the settled solids comprise up to 80 wt% moisture, even after settling process. Such high moisture content makes it difficult for the solids to ignite in the reactor and increases the energy consumption in the reactor due to moisture evaporation, reducing the amount of energy available for recovery. Having varied the desired moisture content in the dried solids, it can be observed that on reduction of the moisture content from 40 to 10 wt% in the drying section of the reactor_,_ the specific net power output of the Stirling engine increases from 12.3 to 14.3 Wh/kg_settledsolids_, respectively, and of the entire system increases from 0 to 1.9 Wh/kg_settledsolids_, respectively (Fig.3a). This is a result of increase in the adiabatic flame temperature in the reactor from 1515.2 °C (40 wt% moisture) to 1805.0 °C at 10 wt% moisture in the dried solids (Fig.3b) due to less energy consumed for moisture evaporation. The drying gas temperature was found to subsequently increase from 1017.8 °C to 1229.7 °C, and thus the amount of energy carried by the drying gas to the drying section increased on reduction of the dried solids moisture content. Yet, the temperature of solids leaving the drying section of the reactor dropped from 159.5 to 144.6 °C, respectively, due to more moisture being removed by the drying gas. Such drop in the temperature of the dry solids entering the reaction chamber may affect their ignition, which is expected to occur at temperatures above 200 °C as the volatile matter is released at similar temperatures from biomass and lignite [13], [42], [43], [44].

**Fig. 4:**
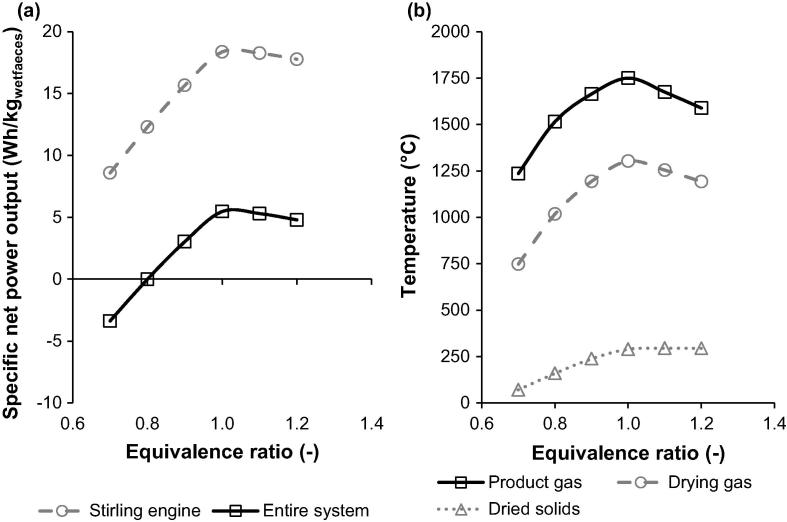
Effect of equivalence ratio on specific power output and temperatures throughout the energy and water recovery system.

To make ignition of dried solids in the reaction zone easier, their temperature, and thus the temperature of the drying gas, needs to be increased. On increase of the air flow rate that corresponds to an increase in the ER from 0.8 to 1.1, the temperature of dried solids (40 wt% moisture) increased from 159.5 °C to 294.6 °C, respectively. A smaller decrease in the dried solids temperature was observed for higher ER values. In addition, the product gas and drying gas temperatures were found to decrease for the ER values above 1 (Fig.4b). This is because more air than the stoichiometric amount required for complete combustion was fed to the reaction chamber, imposing an additional heat requirement to preheat the excess amount of air to the reactor temperature.

Furthermore, an increase of the ER from 0.8 to 1 was found to increase the amount of energy available for recovery, as seen in the rise in the specific net power output of the Stirling engine and the entire system to 18.4 and 5.5 Wh/kg_settledsolids_, respectively. It must be highlighted, however, that for ER values below 1 the chemical energy of solids is not fully utilised, as not all combustibles are fully converted to CO_2_ and H_2_O. It was also found that for the ER values higher than 1, the specific power output decreases, following the decrease in the product gas temperature. Therefore, the proposed should be operated at the ER of 1.1, in order to maximise the dried solids temperature and ensure complete conversion of the settled solids, without compromising the specific net power output.

#### 3.3.2 Energy and water recovery island

**Fig. 5:**
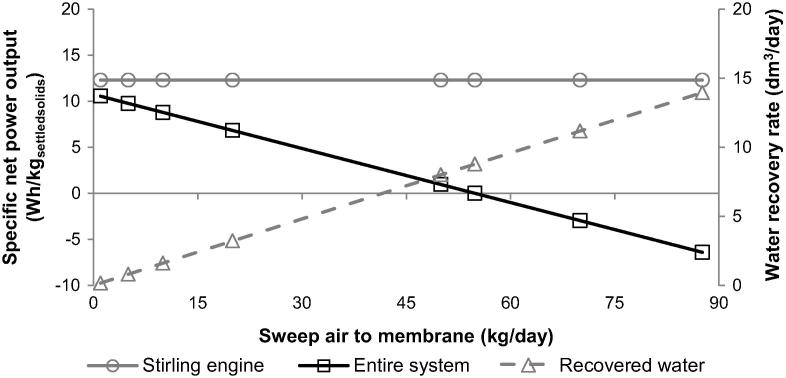
Effect of the amount of sweep air fed to membrane on specific power output and water recovery rate.

While varying both desired moisture content of dried solids and the ER value, the water recovery rate was kept at the initial value of 8.8 dm^3^/day. Yet, it is possible to increase the water recovery efficiency by increasing the amount of the sweep air fed across the membrane (Fig. 5).

The parametric analysis indicated that the amount of water recovered from supernatant increases linearly with the sweep air flow rate. It needs to be highlighted, however, that larger membrane surface area would be required to maintain the desired pressure drop of 4 mbar. It was shown that the maximum water recovery rate of 14.0 dm^3^/day is achievable, which corresponds to 90.9% of the total amount of water fed to the system, if 87.6 kg/day sweep air is fed across the membrane. This is because the remaining part of the recovered water will be carried over with the retentate as water vapour. Importantly, the maximum water recovery rate was found to be higher than the unbound water rate fed to the system (13.9 dm^3^/day), proving that the bound water can be partially recovered from the settled solids. Such improvement in the water recovery level came at an expense of the specific net power output of the entire system (2.5 Wh/dm^3^), making it not self-sustained regardless of unaltered specific power output of the Stirling engine. This was a result of the increased power requirement for increasing the sweep air pressure to overcome the pressure drop across the membrane. The parametric study, therefore, revealed a trade-off between the amount of water that can be recovered from supernatant and the system’s power output that can be utilised elsewhere. Therefore, an increase in the water recovery efficiency can be achieved on increase of the specific power output of the entire system and its utilisation to drive the membrane process.

**Fig. 6:**
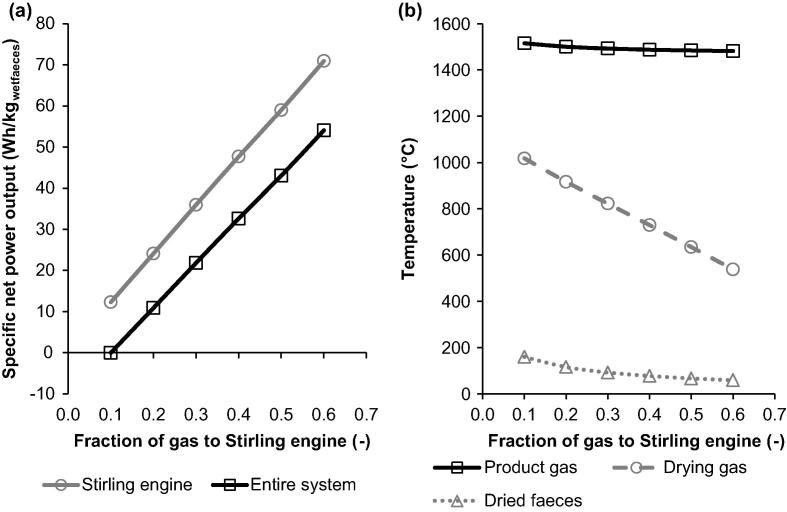
Effect of fraction of gas directed to Stirling engine on specific net power output and temperatures throughout the energy recovery system.

The initial design basis assumed that the area of the Stirling engine heater is sized for 10% mass flow rate of gas leaving the reactor, limiting the amount of energy that is utilised for power generation. An increase in the area of the Stirling engine heater, which corresponds to subsequent increase of the fraction of gas fed to this heater from 10% to 60%, is found to substantially increase the specific net power output of the entire system from 0 to 54.1 Wh/kg_settledsolids_ (Fig.6a), with the water recovery rate kept at 8.8 dm^3^/day. However, it was observed that the more energy is utilised for power generation in the Stirling engine, the lower the drying gas temperature is (Fig.6b). As a result, a substantial decrease in the temperature of the dried solids is observed, what would have an effect on its ignitability in the reactor. Based on the considerations made above, the amount of energy utilised in the Stirling engine for power generation should be only increased, if the resulting dried solids temperature of at least 200 °C can be maintained.

**Fig. 7:**
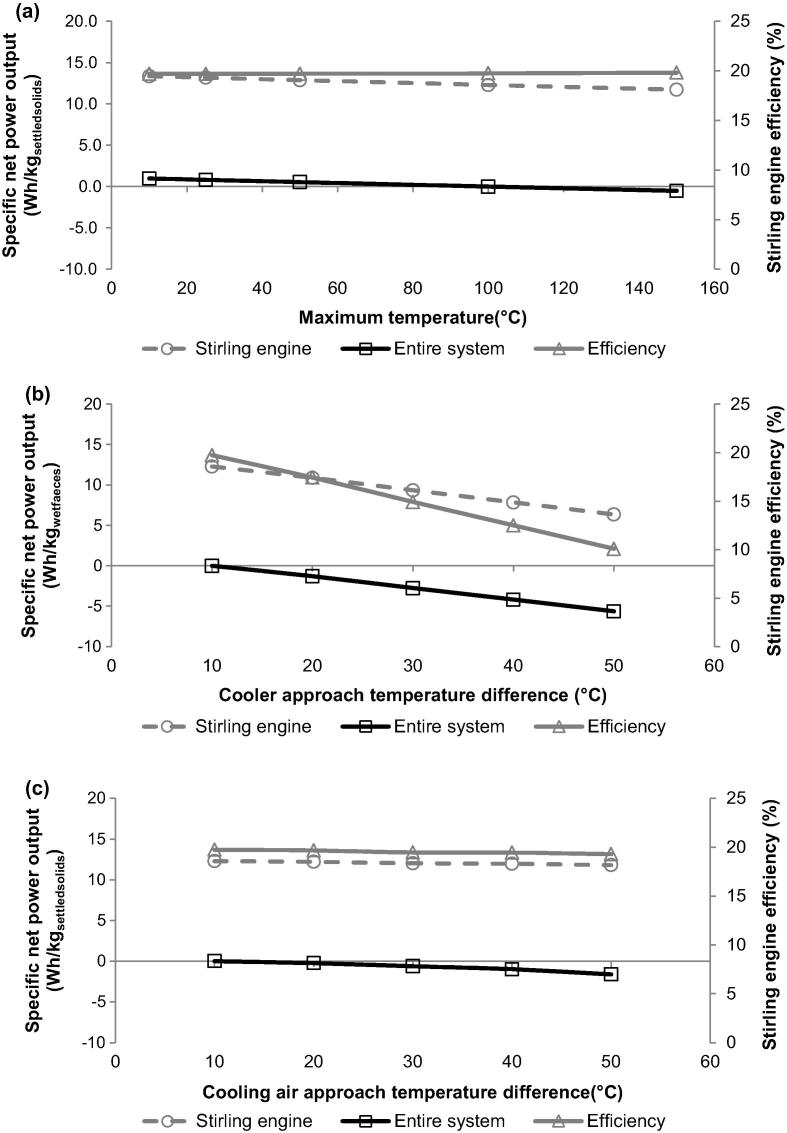
Effect of the Stirling engine cooling air approach temperature difference on specific net power output and efficiency of the Stirling engine.

Importantly, the specific net power output of the entire system is directly dependent on the operating temperature envelope of the Stirling engine that influences its efficiency. As indicated above, the Stirling engine operates with the efficiency of 19.7% at the initial design conditions, which assume the approach temperature differences of 100, 10 and 10 °C for the Stirling engine heater, cooler and cooling air, respectively, and the maximum temperature of the working medium of 600 °C. Fig.7 reveals that only a change in the cooler approach temperature difference (from 10 to 50 °C) has a meaningful effect on the efficiency of the Stirling engine, reducing it from 19.8 to 10.1%. In general, it was found that increase in the approach temperature difference results in drop in the specific power output of the entire system, mostly due to less heat transferred to the working medium in the heater (Fig.7a) or removed from the working medium in the cooler (Fig.7b), as well as more air required to achieve desired cooling duty in the cooler (Fig.7c) that increases power requirement of the air fan. Nevertheless, an increase in the specific power output of 0.98 Wh/kg_settledsolids_ can be only achieved by reducing the approach temperature difference of the heater from 100 to 10 °C, yet at expense of large heater area. Therefore, the optimum approach temperature differences of the heat exchangers should be selected via techno-economic analysis with an objective to maximise the process performance and minimise the systems’ capital cost.

**Fig. 8:**
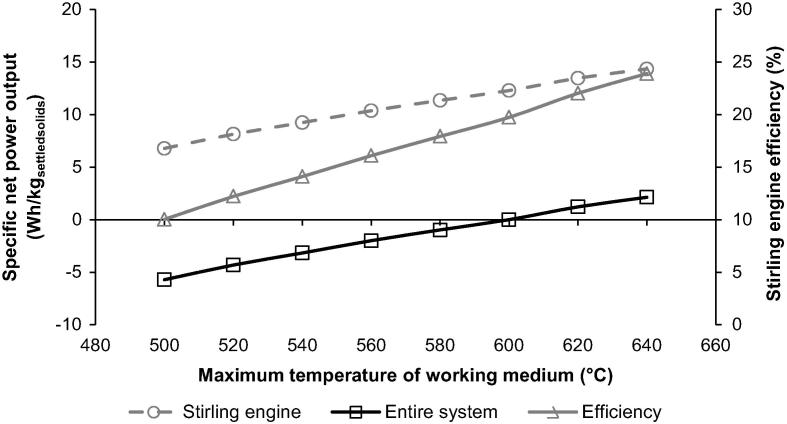
Effect of maximum operating temperature of the Stirling engine on specific net power output and efficiency of the Stirling engine.

Finally, the efficiency of each thermodynamic cycle is highly dependent upon the maximum temperature of the medium in the cycle, and can be increased on temperature increase [45], [46]. By varying the maximum temperature of working medium in the Stirling engine between 500 and 640 °C, the cycle efficiency changed between 10.0 and 23.9%, while the water recovery rate was kept at 8.8 dm^3^/day. This in good agreement with the efficiency range of 6–27% reported in the literature for the Stirling engines operating under different operating conditions [38], [39], [40], [41], [47], [48]. It needs to be highlighted that the efficiency of the Stirling engine and the net specific power output of the entire system increased by 4.2% points and 2.1 Wh/kg_settledsolids_, respectively, on increase in the maximum temperature of the working medium from 600 to 640 °C (Fig. 8).

### 3.4 Process performance under revised design basis

Considering the findings from the parametric study, the design basis presented in Section 2.2 is revised to maximise the process performance. First, it is assumed that the temperature of the dried solids fed to the reactor is at least 200 °C to ensure a continuous process. Second, to maximise the amount of recoverable energy, the reactor operates under the combustion regime, with the ER value of 1.1 to maximise the process performance and to keep the temperature of the dried solids at a desired level. This allowed increasing the fraction of gas to Stirling engine from 10 to 18.7%. Third, the maximum temperature of the working medium in the Stirling engine is increased from 600 to 640 °C, and the heater approach temperature difference is reduced from 100 to 25 °C. Although the latter will require the heat exchanger of larger surface area, it is expected that the performance gain will outweigh the economic burden. Finally, the desired moisture content in the dried solids fed to the reactor is reduced to 20 wt%.

The performance analysis (Table 4) of the conceptual energy and water recovery system for the Nano Membrane Toilet operating in the nominal operating mode (maximum water mode) indicates that the system is able to recover 87.0% of the total amount of water fed to the system and deliver the net power output of 1.9 W, which corresponds to the specific net power output of 23.1 Wh/kg_settledsolids_. It needs to be highlighted that as a result of increased mass flow rate of gas leaving the dryer, which is a result of higher ER value, more bound water leaves the system with that gas stream. As a result, the water recovery rate is slightly lower compared to the maximum figure identified in Section 3.3.2 (14.0 dm^3^/day). Nevertheless, the power output of the proposed concept is comparable to 2.5 W of the USB port peak power (5 V, 500 mA) [49], indicating that it can be utilised for charging of electronic devices, such as mobile phones, or provide light for the household using low-voltage 2 W LED bulbs [50]. Importantly, the net power output of the system can be increased when less supernatant is preheated and the water recovery rate is compromised. The system operating in the power generation mode (maximum power mode) will deliver the net power output of 5.8 W, which corresponds to the specific net power output of 69.2 Wh/kg_settledsolids_ and is comparable to average power consumption of the mobile phone chargers, or even clock radios [51].

This performance is worse than in the system proposed by Liu et al. [14], which was characterised with the net power output of 194.4–357.3 Wh/kg_settledsolids_. However, it needs to be highlighted that the proposed by Liu et al. [14] utilises plasma gasification technology that is only in an early development stage and solid oxide fuel cells that are expensive, and thus has not been practically utilised at domestic or industrial scale [52]. Conversely, the concept proposed in this study utilises the components that are commercially available at a considerably lower capital cost compared to plasma gasification and solid oxide fuel cells. Despite the fact that the efficiency of the solid oxide fuel cells can be approximately three times higher than the efficiency of the Stirling engine considered in this study [53], commercial deployment the former system is unlikely within reasonable timeframe. Implementation of the conventional gasification, which was found viable for gasification of livestock manure [23], [54], in an integrated gasification combined cycle power plant may be a more reasonable option [55]. Moreover, the human waste to power system proposed by Liu et al. [14] did not consider the supernatant processing and was designed to process around 69 kg of the settled solids from human excreta per day. This figure is considerably higher than 2 kg of the settled solids from human excreta per day for a household of ten people. Therefore, the system proposed by Liu et al. [14] can be considered as a community, rather than household scale unit. Furthermore, the commercial maturity of the key components in the proposed system and the relevant maintenance experience indicate that the Nano Membrane Toilet would have low maintenance requirement. Namely, the nano membrane is characterised by low maintenance requirements, providing that the air fan system is properly maintained to ensure no solids and water in the sweep air that could reduce the nano membrane performance and operating lifetime [56], [57], which is expected to be up to ten years. It is predicted that the nano membrane would require regular cleaning, in example every three months. The wet faecal matter dryer is expected to have low maintenance requirement if the system is operated with relatively low oxygen concentration in the flue gas to avoid self-ignition, and thus fire risk in the dryer [58]. Similarly, the Stirling engine is regarded as maintenance-free or low-maintenance, and long-lifetime unit with a routine replenishment of working fluid being the main requirement [59], [60]. Finally, the flawless operation of the reactor would require low-maintenance that can be ensured by regular activities, such as visual inspections, emptying the ash bin, which can be used as fertiliser, and greasing of air [61]. Therefore, similarly to the conventional flush toilet, the key maintenance activities required by the Nano Membrane Toilet would be regular inspection and cleaning.

As storage of recovered water would be cheaper than storage of electricity, which also incurs additional energy conversion losses [62], it is expected that the system operating in the nominal operating mode (maximum water mode) will be characterised with lower costs compared to the power generation mode (maximum power mode). Therefore, the latter operating mode should be used only during increased energy demand periods. Importantly, the performance of conceptual energy and water recovery system for the Nano Membrane Toilet proposed in this study can be further improved by a detailed heat exchanger network analysis and incorporation of the optimisation algorithms. Yet, this needs to be conducted along with the economic assessment of the proposed system, which is out of the scope of this study and currently under investigation by the authors, in order to maximise its techno-economic performance.

## 4 Conclusions

In this study, the thermodynamic performance of the conceptual energy and water recovery system for the Nano Membrane Toilet was evaluated. The process model that comprises the thermochemical conversion island, and energy and water recovery island was modelled in Aspen Plus®. The former utilises the Aspen Plus® solid modelling features to represent the thermochemical conversion of the settled solids from human excreta using an equilibrium approach. The latter includes the heat exchanger network model for supernatant preheating and the pseudo Stirling engine model for electricity production. Moreover, the model accounts for the power requirement to overcome the pressure loss throughout the system.

Evaluation of the process performance revealed that under the initial design conditions, the conceptual energy and water recovery system is capable of producing enough electricity for the entire system to become self-sustained and to recover 8.8 dm^3^ of water a day. As this was found to be only 57.1% of the amount of supernatant fed to the system, a further parametric study was undertaken to maximise power output and water recovery rate. This study revealed that performance of the specific power output of the entire system can be increased by reducing the moisture content in the dried solids fed to the reactor. Yet, as the temperature of dried solids leaving the dryer was found to decrease with the amount of moisture removed, what is expected to have a negative effect on the volatiles release and thus their ignition in the reactor. This effect could be overcome by operating the reactor with higher ER values. Importantly, the parametric study revealed a trade-off between the power output and the rate of water recovery, in addition to the optimal design conditions. In the nominal operating mode (maximum water mode), the net power output of the entire system was estimated to be 1.9 W, which corresponds to the specific net power output of 23.1 Wh/kg_settledsolids,_ and the water recovery rate was 13.4 dm^3^/day. Such net power output is comparable to 2.5 W of the USB port peak power (5 V, 500 mA), indicating that it can be utilised for charging of electronic devices, such as mobile phones, or provide light for the household using low-voltage 2 W LED bulbs. In case of increased power demand, the net power output of the system can be increased up to 5.8 W, which corresponds to the specific net power output of 69.2 Wh/kg_settledsolids_, by compromising the water recovery rate. Such net power output is comparable to average power consumption of the mobile phone chargers or clock radios. Therefore, the proposed system can provide an alternative source of electricity and/or water for these communities.

## Figures and Tables

**Table 1:** Human excreta composition.

Settled solids [3]		Supernatant [33]	
Component	As received (wt%)	Component	As received (wt%)
*Proximate analysis*		*Mass concentration*	
Fixed carbon	7.26	Water	97.16
Volatile matter	11.72	Urea	1.38
Ash	3.99	Sodium chloride	0.82
Moisture	77.03	Potassium chloride	0.17
	Dry ash free (wt%)	Potassium sulphate	0.27
*Ultimate analysis*		Magnesium sulphate	0.08
Carbon	61.52	Magnesium carbonate	0.01
Hydrogen	8.23	Potassium bicarbonate	0.07
Oxygen	25.31	Lysine	0.01
Nitrogen	4.95	Asparagine	0.01
		Phenol	0.03

**Table 2:** Initial design conditions for the conceptual energy and water recovery system.

Parameter	Value
Equivalence ratio (–)	0.8
Specific power requirement for screw conveyor (J/kg_settledsolids_)	200
Isentropic efficiency of air fan (%)	90.0
Mechanical efficiency of air fan (%)	99.8
Desired moisture content of dried solids (wt%)	40
Air preheater approach temperature (°C)	10
Membrane sweep air approach temperature (°C)	10
Vapour fraction in retentate (%)	80
Supernatant outlet temperature (°C)	60
Maximum temperature of working medium in Stirling engine (°C)	600
Stirling engine heater temperature approach (°C)	100
Stirling engine cooler temperature approach (°C)	10
Stirling engine cooling air temperature approach (°C)	10
Stirling engine regenerator approach temperature (°C)	5
Fraction of gas to Stirling engine (–)	0.1

**Table 3:** Performance indicators of the conceptual energy and water recovery system.

Parameter	Value
*System’s capacity and water input*	
Number of people	10
Settled solids mass flow rate (kg/day, wet basis)	2.0
Supernatant volumetric flow rate from the settling tank (dm^3^/day)	14.6
Unbound water rate (dm^3^/day)	13.9
Bound water rate (dm^3^/day)	1.5

*Key performance indicators*	
Adiabatic flame temperature (°C)	1515.2
Drying gas temperature (°C)	1017.8
Dried solids temperature (°C)	159.5
Stirling engine net power output (W)	1.0
Stirling engine efficiency (%)	19.7
Stirling engine specific power output (Wh/kg_settledsolids_)	12.3
System net power output (W)	0.0
Water recovery rate (dm^3^/day)	8.8
Water recovery level (%)	57.1

**Table 4:** Performance indicators of the revised conceptual energy and water recovery system.

Parameter	Maximum water mode	Maximum power mode
*System’s capacity*		
Number of people	10	10
Settled solids mass flow rate (kg/day, wet basis)	2	2
Supernatant volumetric flow rate (dm^3^/day)	14.6	0.0

*Key performance indicators*		
Adiabatic flame temperature (°C)	1809.2	1809.2
Drying gas temperature (°C)	1248.0	1428.8
Dried solids temperature (°C)	200	200
Stirling engine net power output (W)	3.6	6.2
Stirling engine specific net power output (Wh/kg_settledsolids_)	43.0	74.8
Stirling engine efficiency (%)	23.1	23.1
System net power output (W)	1.9	5.8
Specific net power output (Wh/kg_settledsolids_)	23.1	69.2
Water recovery rate (dm^3^/day)	13.4	0.0
Water recovery level (%)	87.0	0.0
